# Supplementary material for: Altered Phenotypes of Breast Epithelial × Breast Cancer Hybrids after ZEB1 Knock-Out
Source: Int J Mol Sci. 2023 Dec 9;24(24):17310. doi: 10.3390/ijms242417310 (PMC10744253; doi:10.3390/ijms242417310)

# original data – Western Blots ZEB1

**chemiluminescence** | **blot photo** | **overlay**

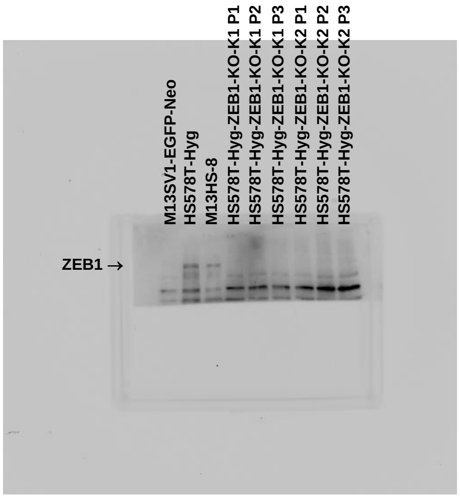


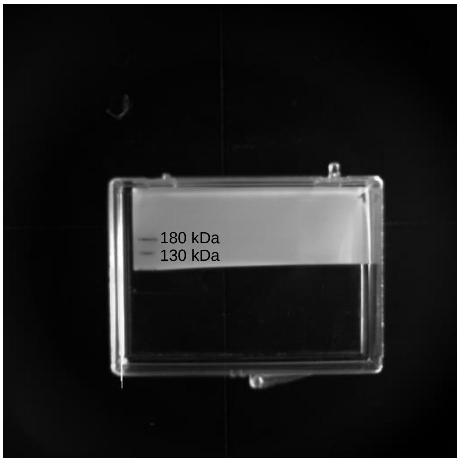


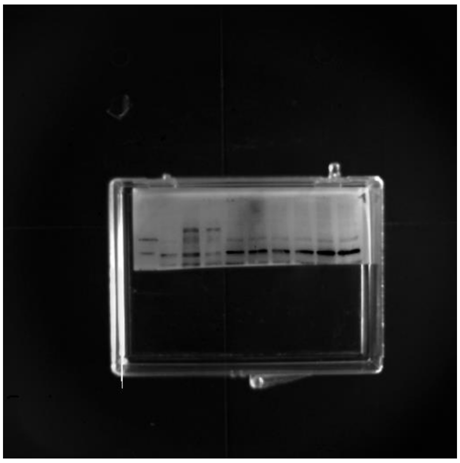

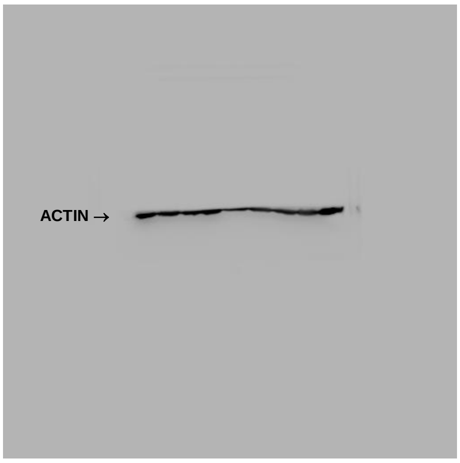


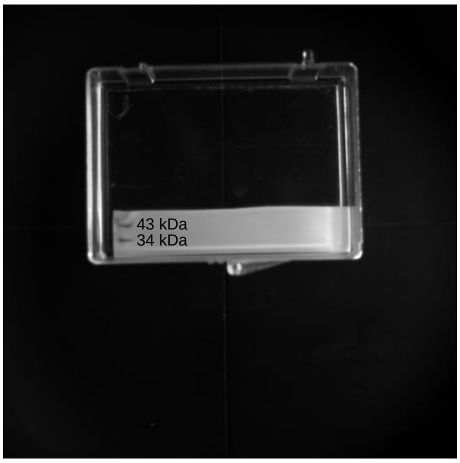


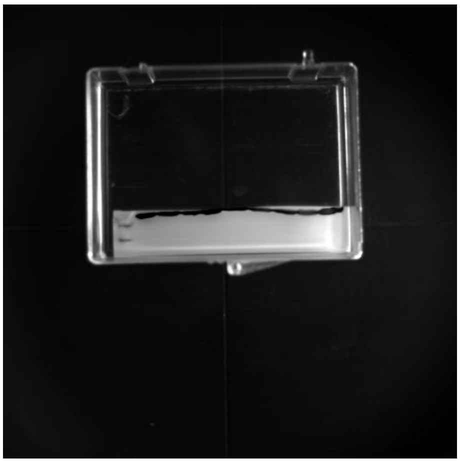

# original data – Western Blots ZEB1

chemiluminescence | blot photo | overlay

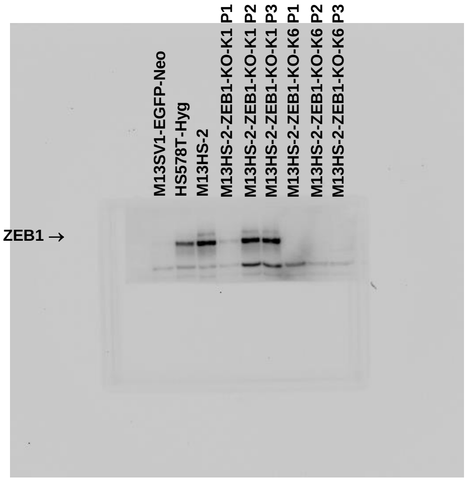


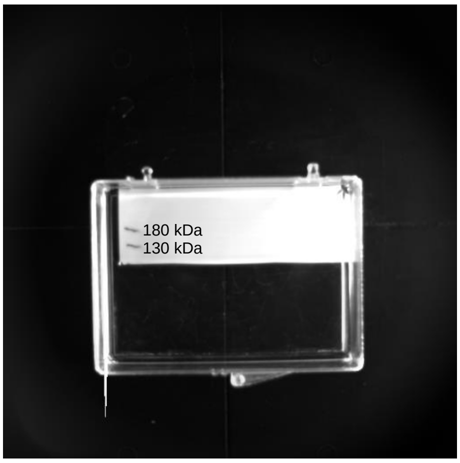


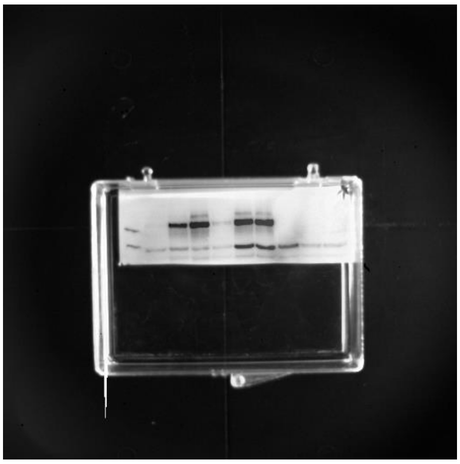

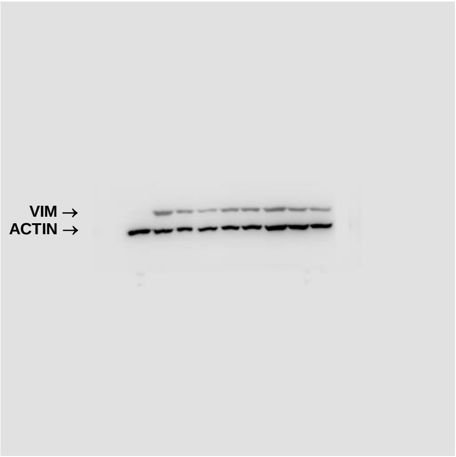


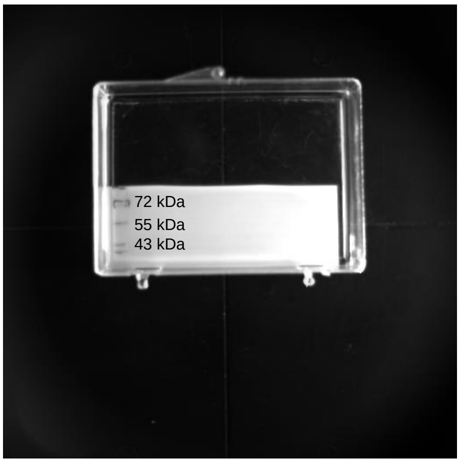


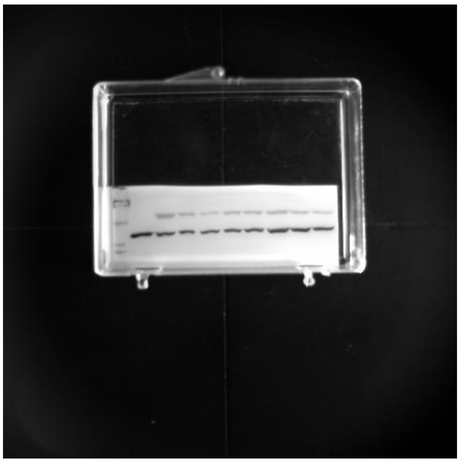

# original data – Western Blots ZEB1

**chemiluminescence** | **blot photo** | **overlay**

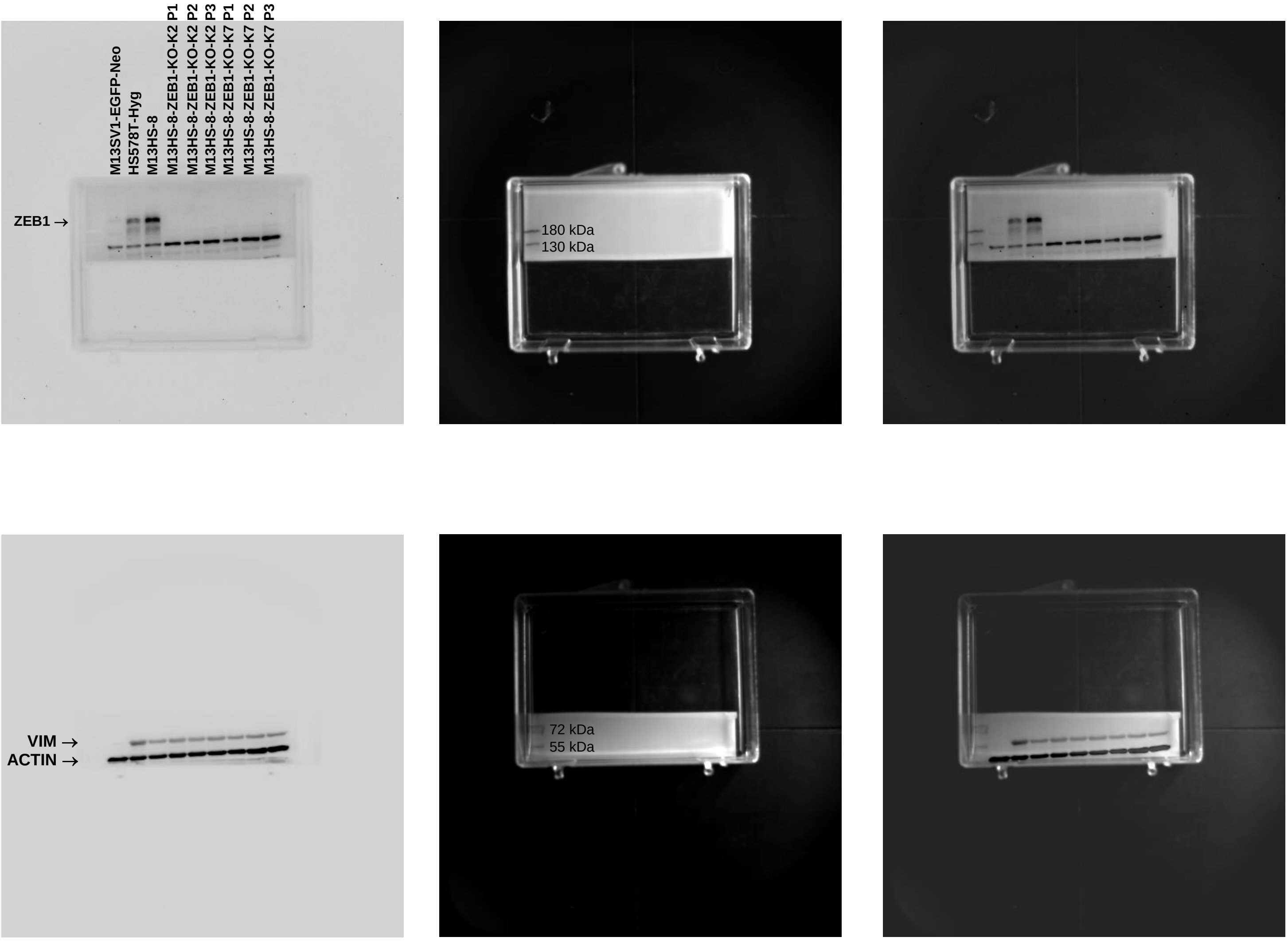

# original data – Western Blots SNAIL

**chemiluminescence** | **blot photo** | **overlay**

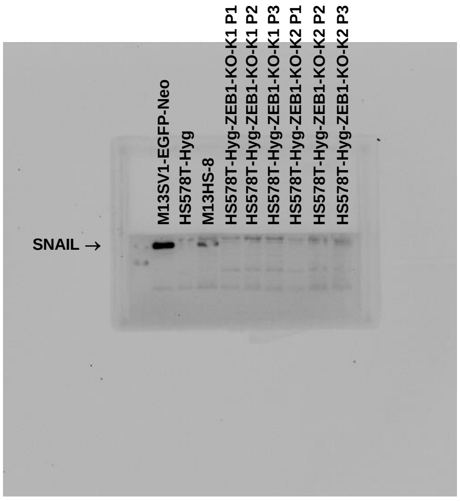


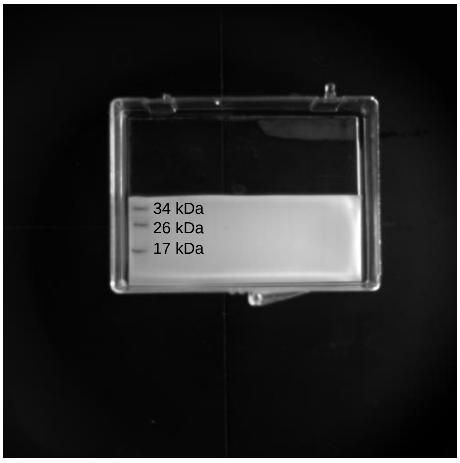


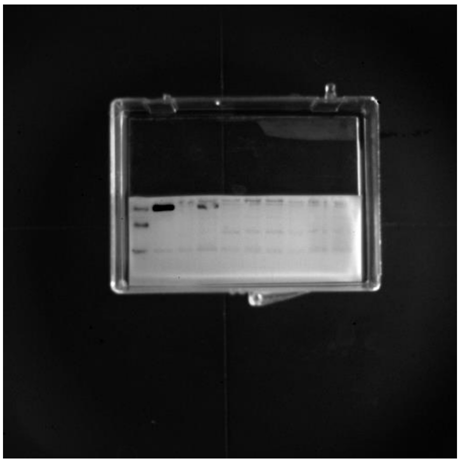

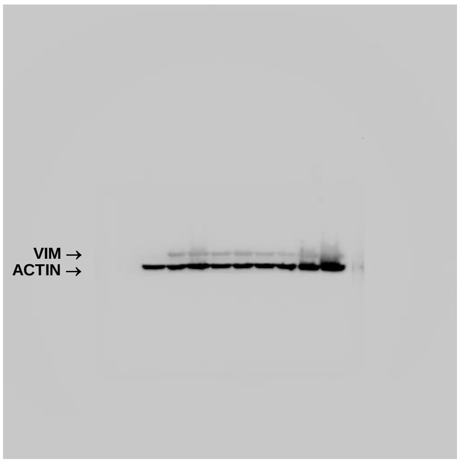


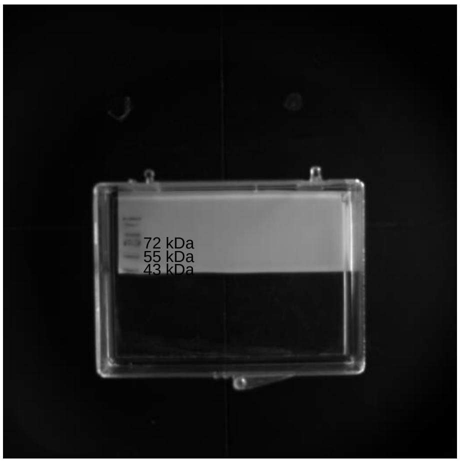


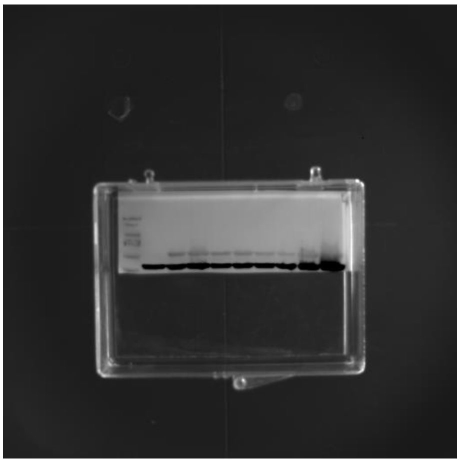

# original data – Western Blots SNAIL

**chemiluminescence** | **blot photo** | **overlay**

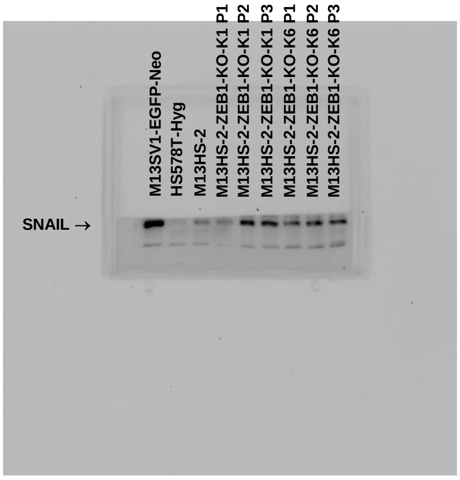


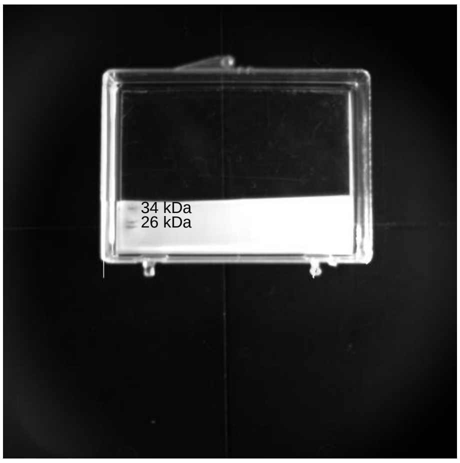


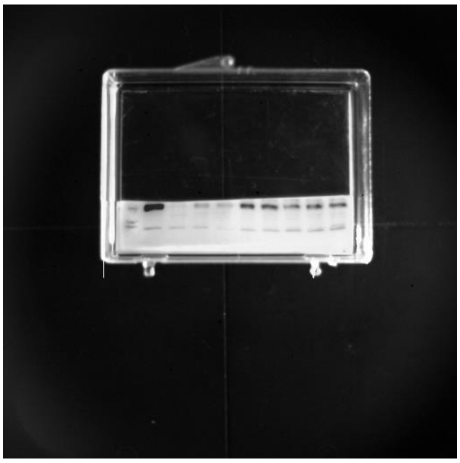

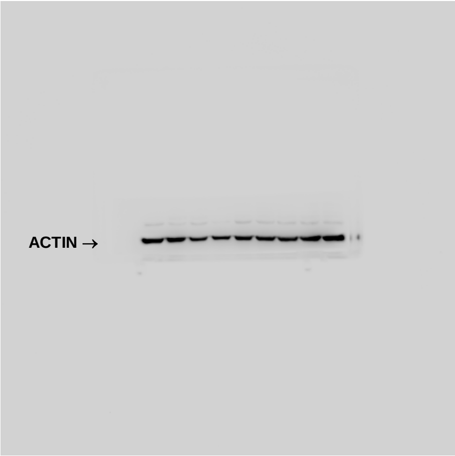


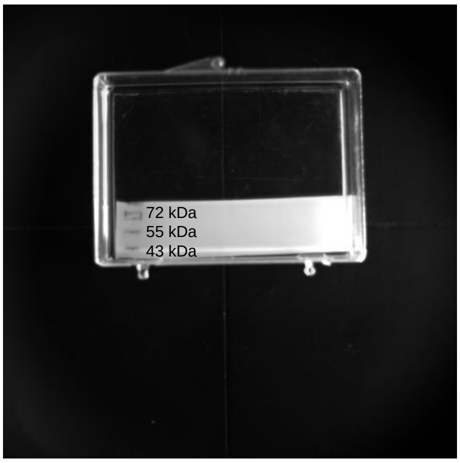


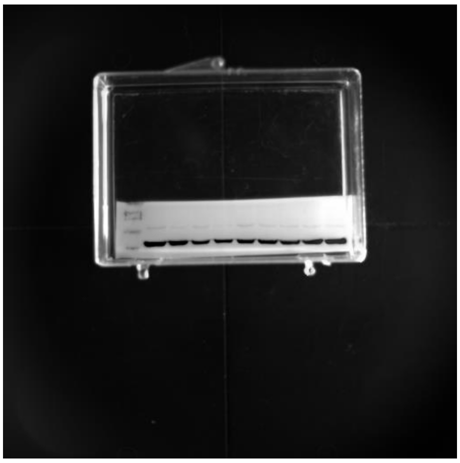

# original data – Western Blots SNAIL

| chemiluminescence | blot photo | overlay |
| --- | --- | --- |

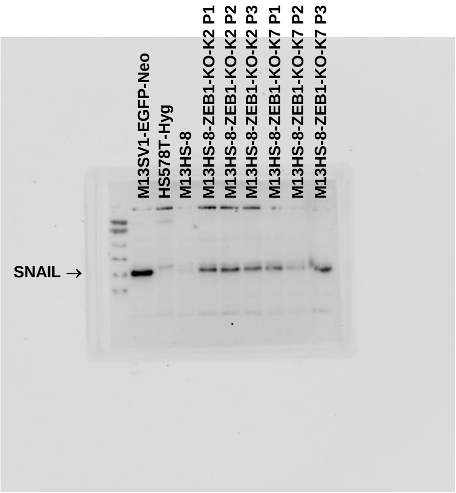


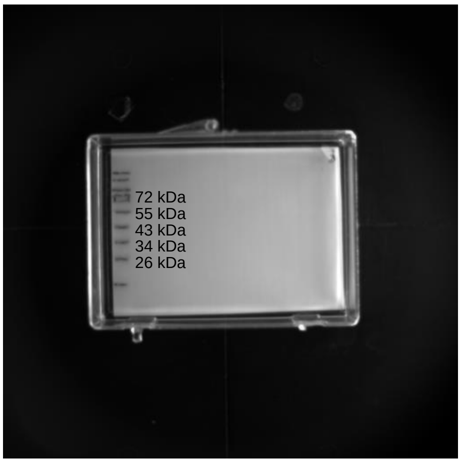


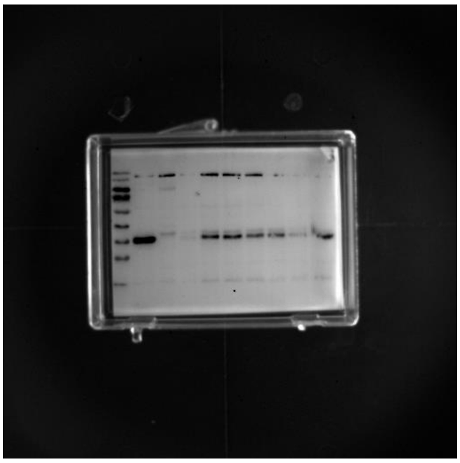

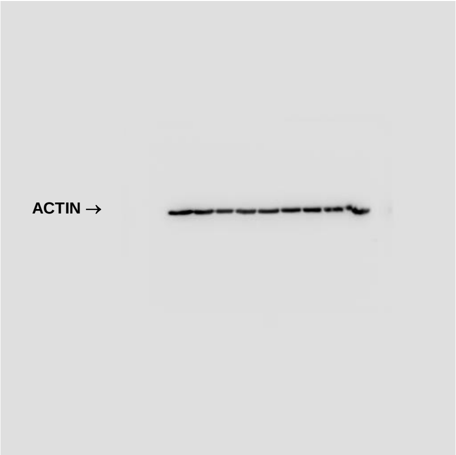


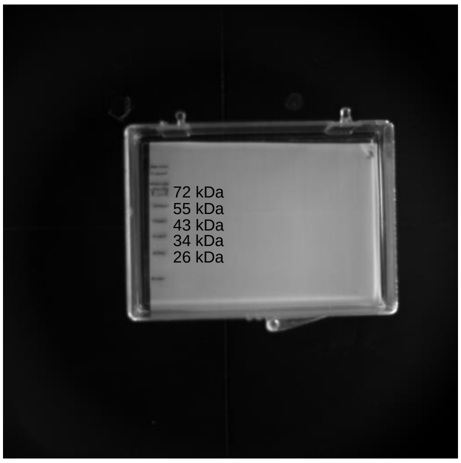


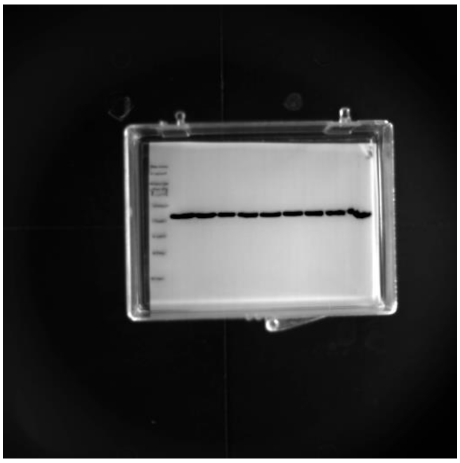

# original data – Western Blots E-CADHERIN

**chemiluminescence** | **blot photo** | **overlay**

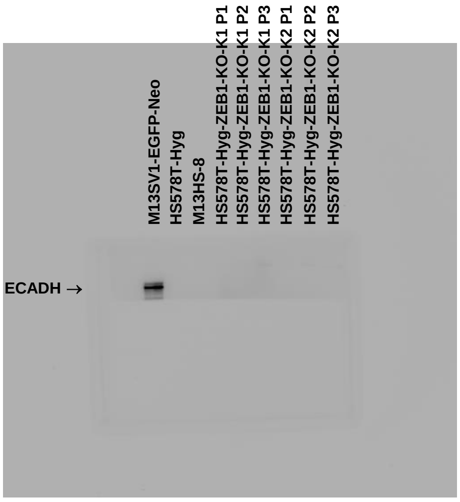


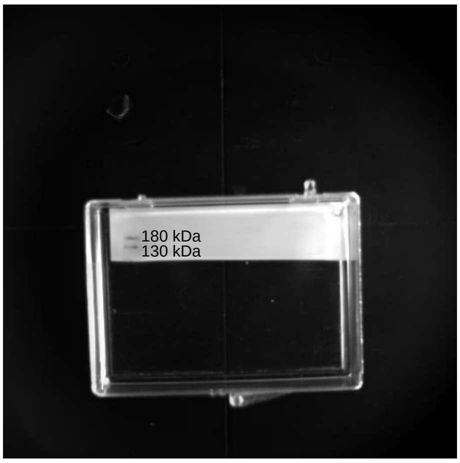


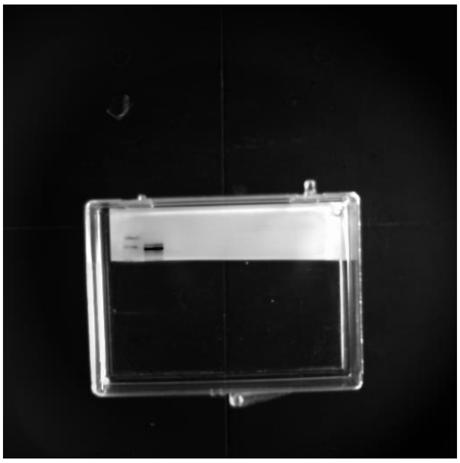

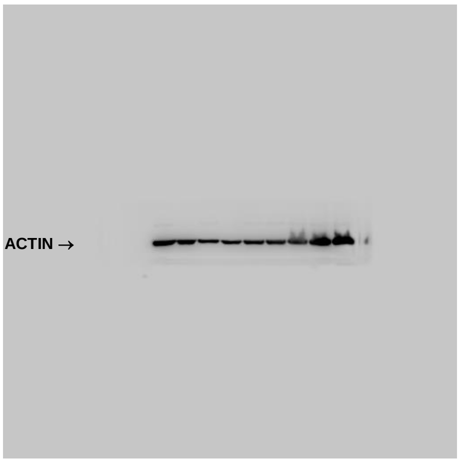


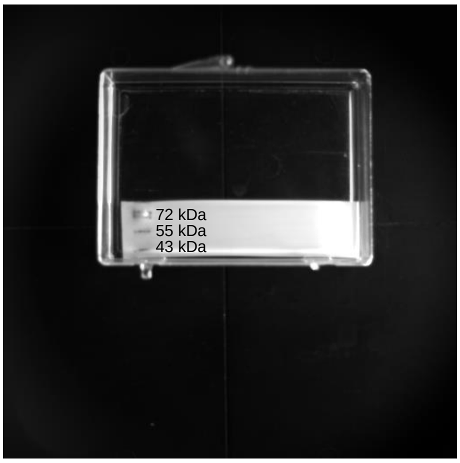


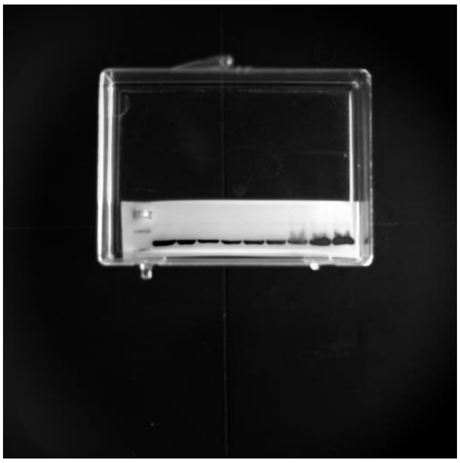

# original data – Western Blots E-CADHERIN

**chemiluminescence** | **blot photo** | **overlay**

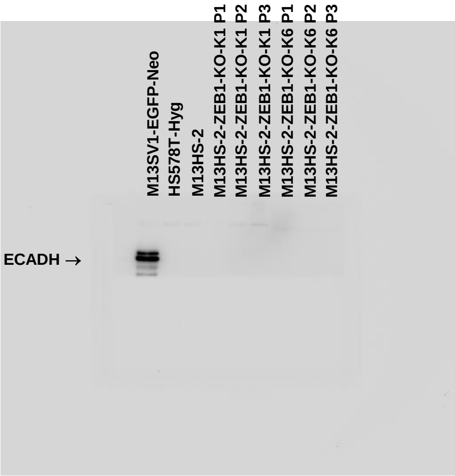


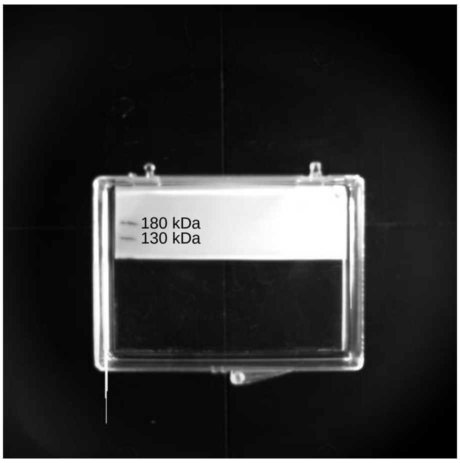


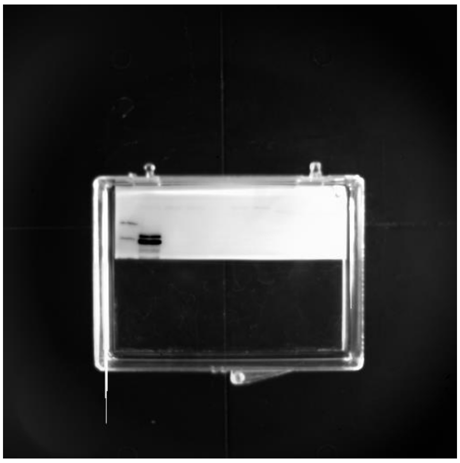

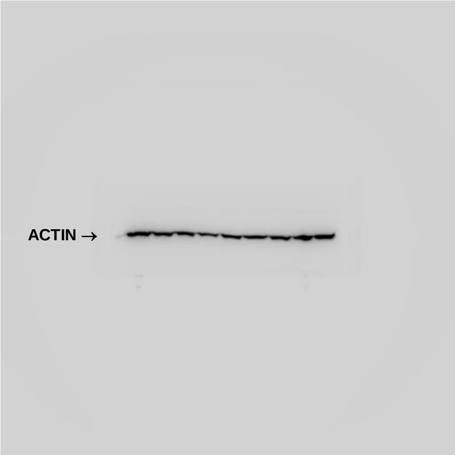


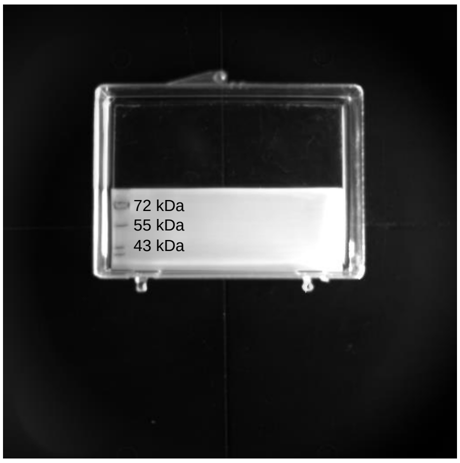


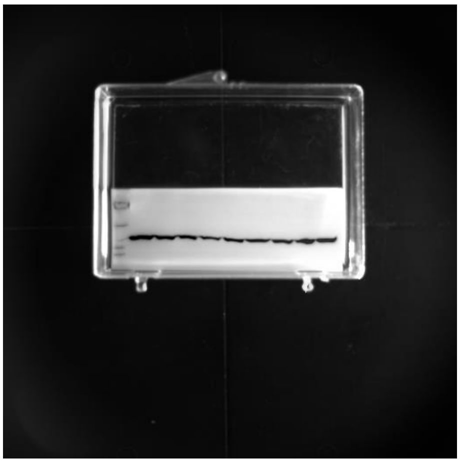

# original data – Western Blots E-CADHERIN

**chemiluminescence** | **blot photo** | **overlay**

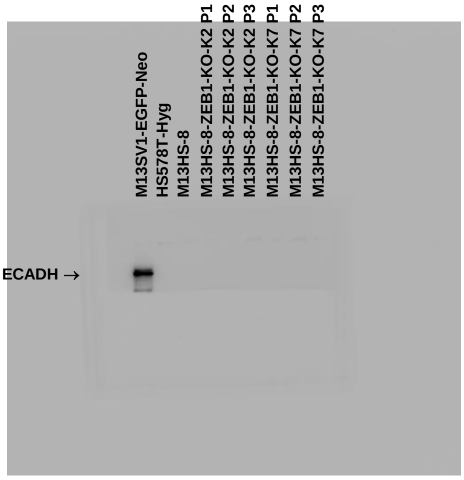


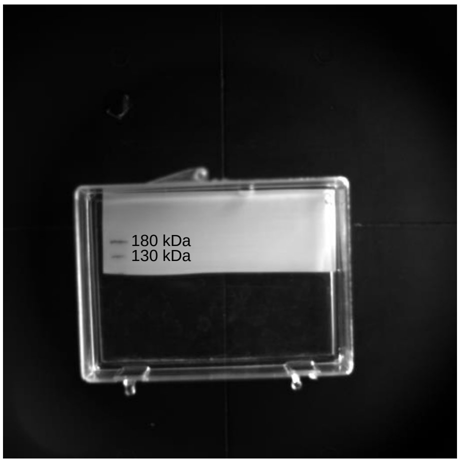


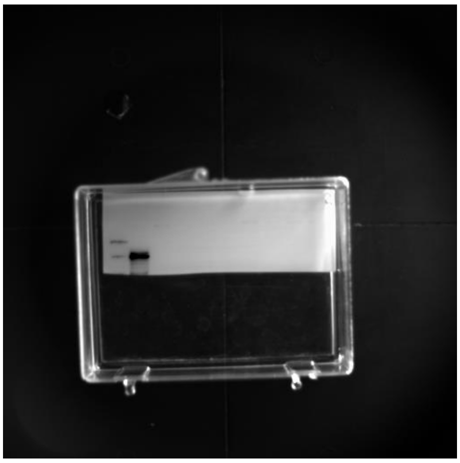

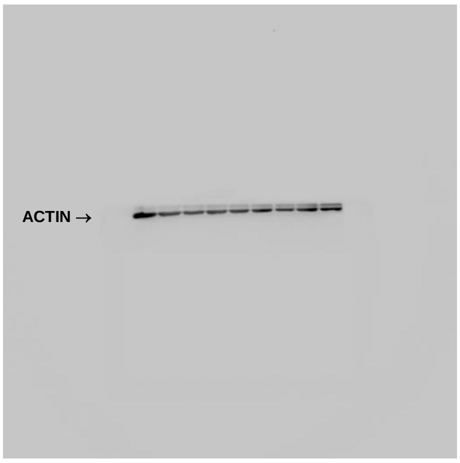


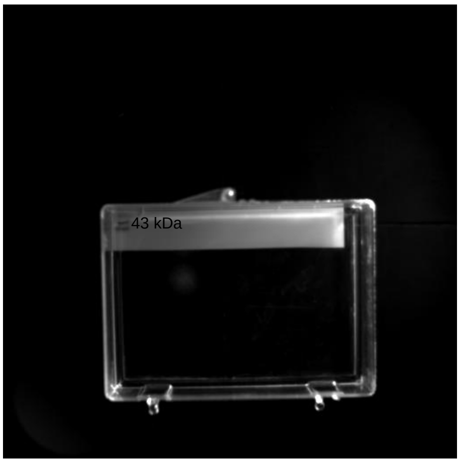


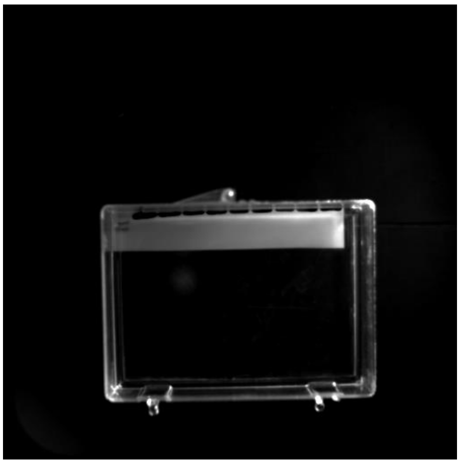

# original data – Western Blots CYTOKERATIN-5

**chemiluminescence** | **blot photo** | **overlay**

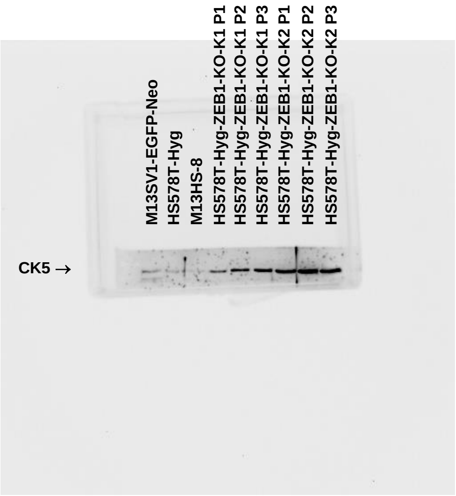


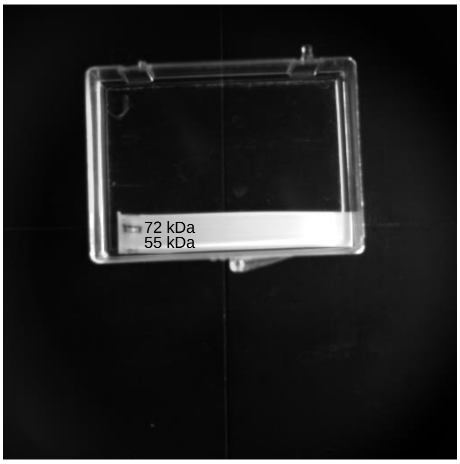


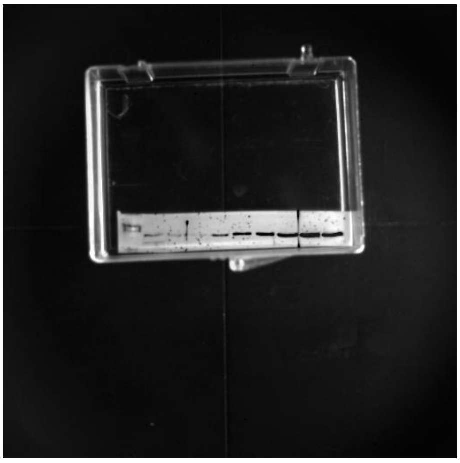

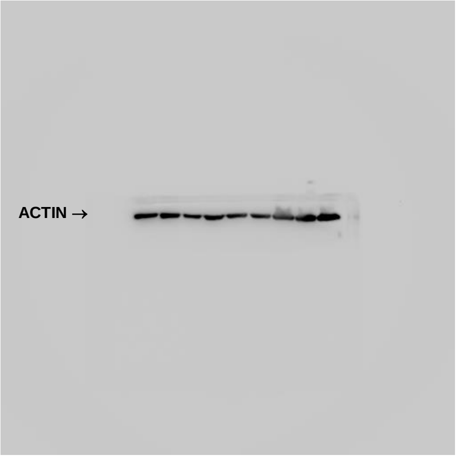


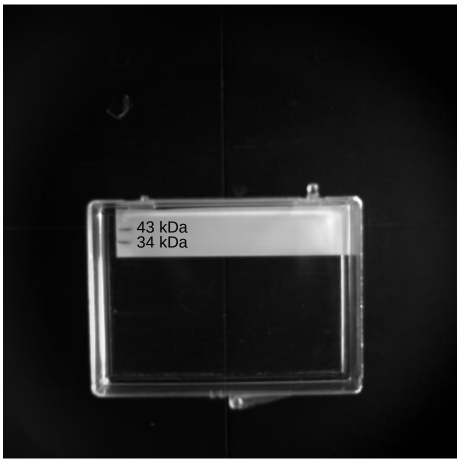


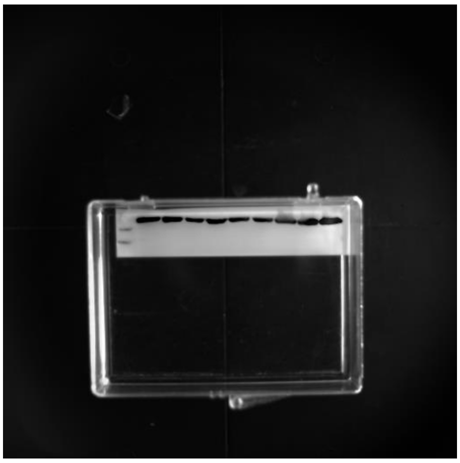

# original data – Western Blots CYTOKERATIN-5

**chemiluminescence** | **blot photo** | **overlay**

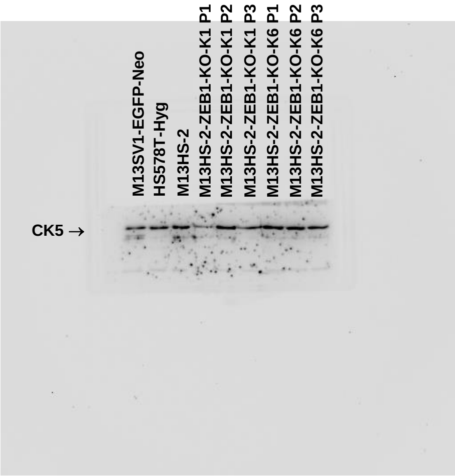


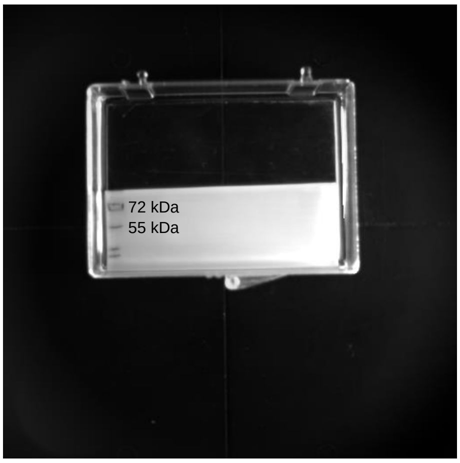


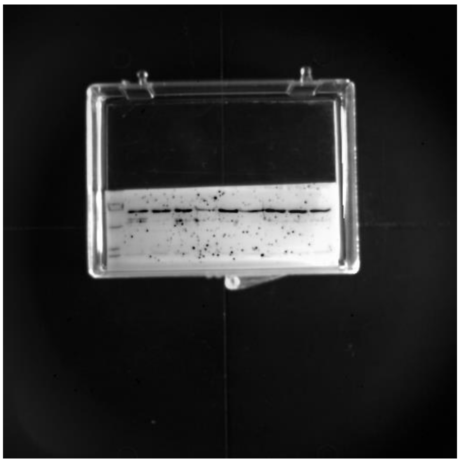

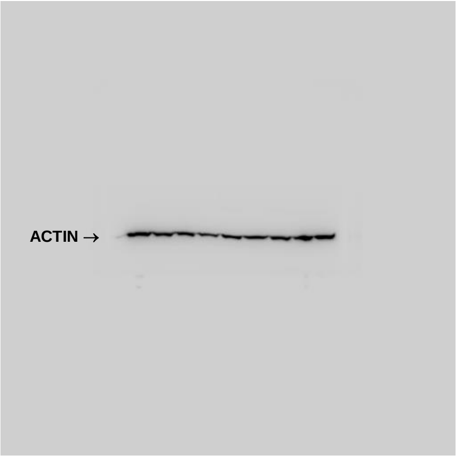


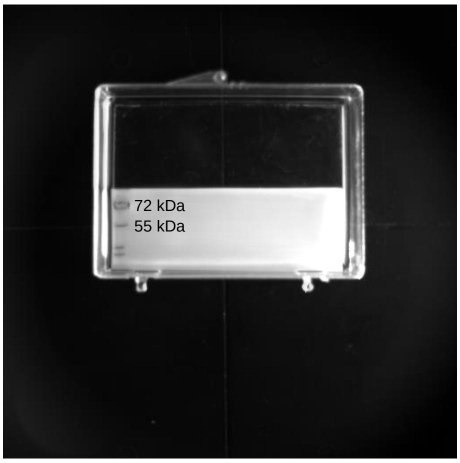


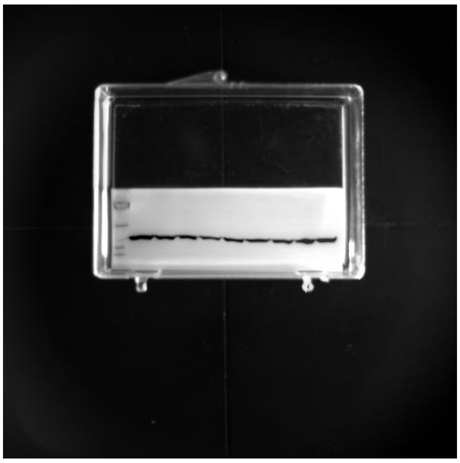

# original data – Western Blots CYTOKERATIN-5

| chemiluminescence | blot photo | overlay |
| --- | --- | --- |

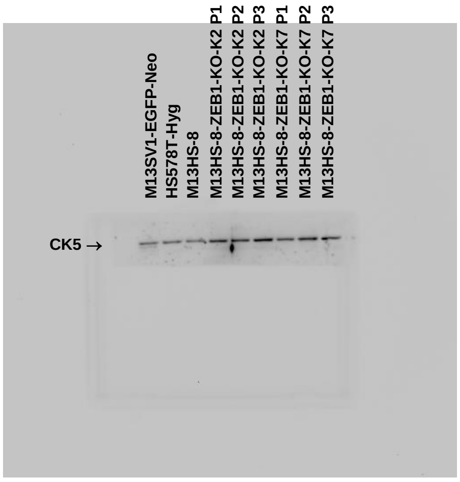


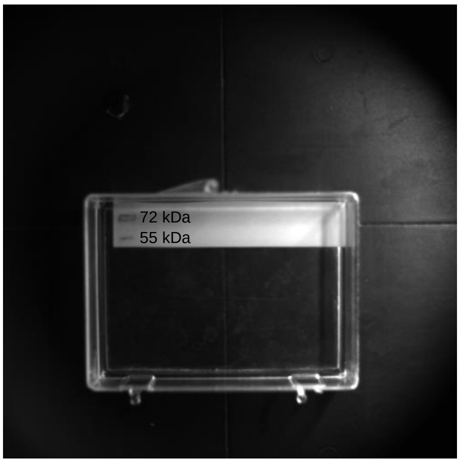


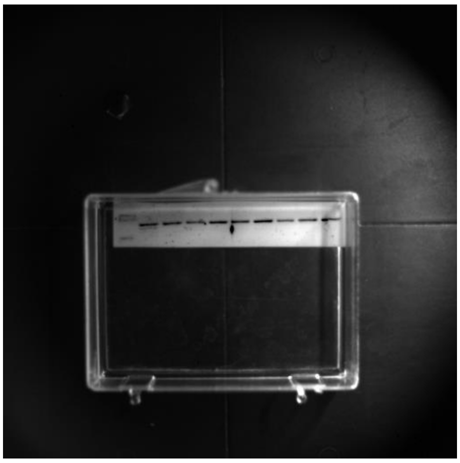

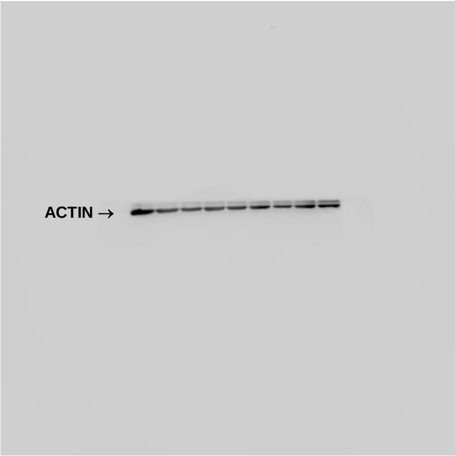


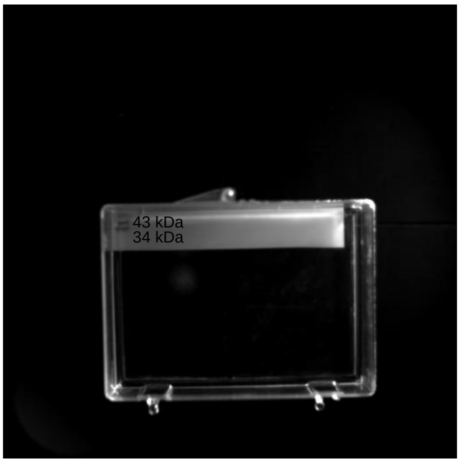


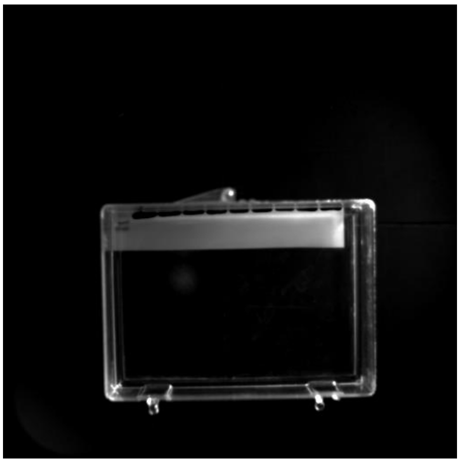

# original data – Western Blots N-CADHERIN

**chemiluminescence** | **blot photo** | **overlay**

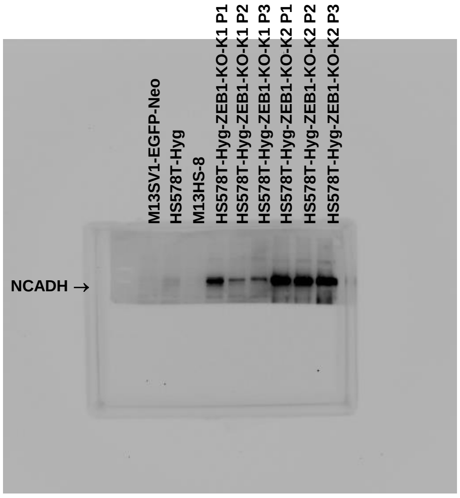


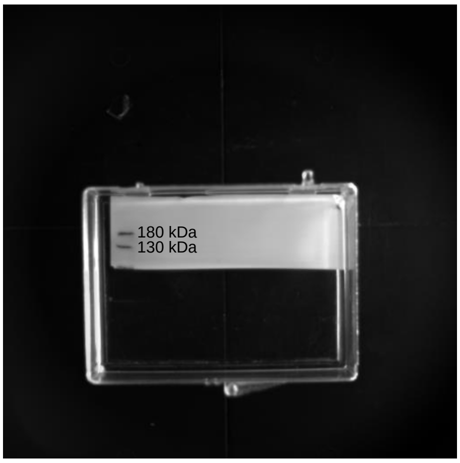


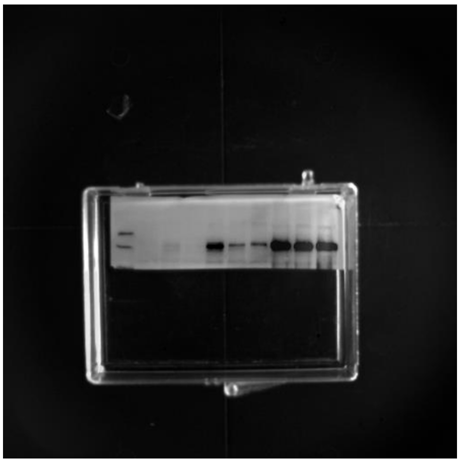

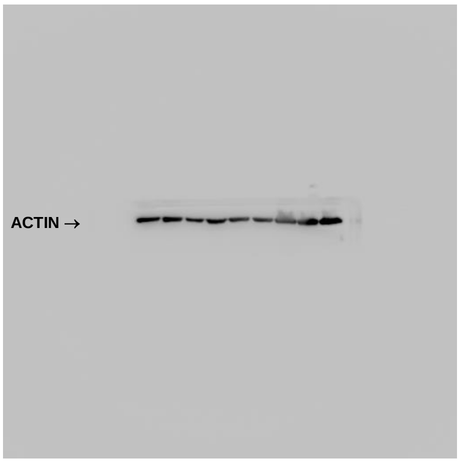


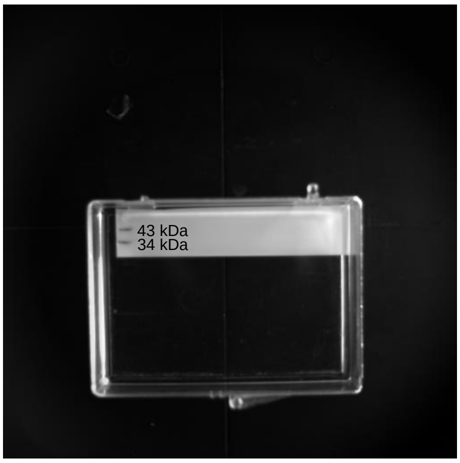


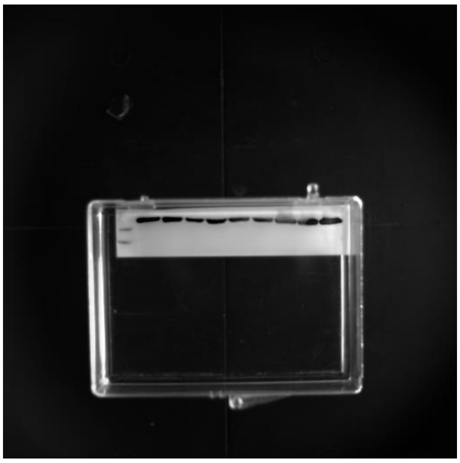

# original data – Western Blots N-CADHERIN

**chemiluminescence** | **blot photo** | **overlay**

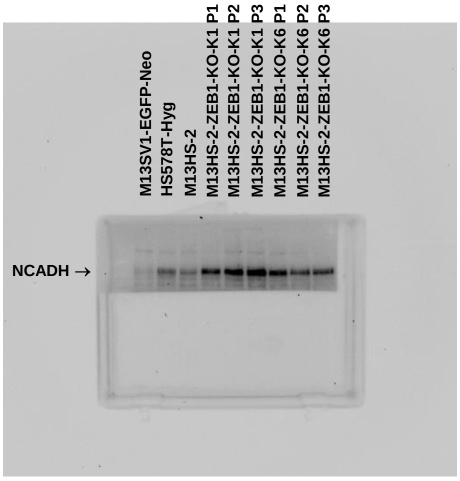


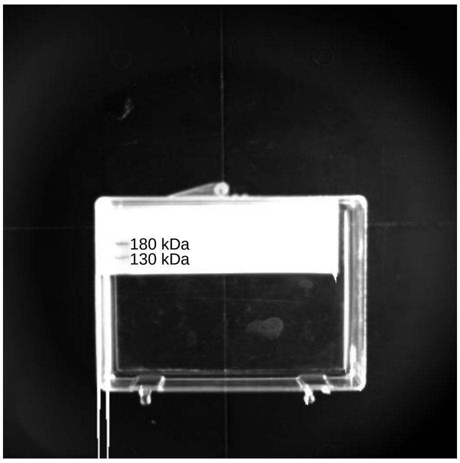


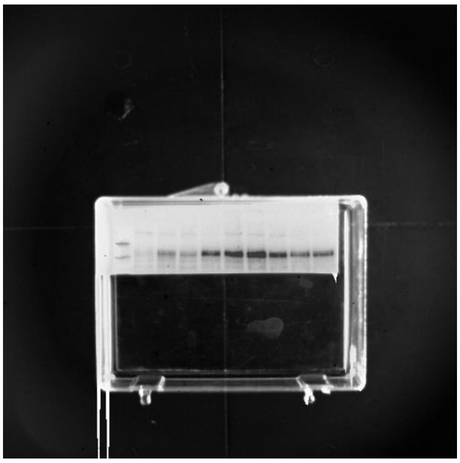

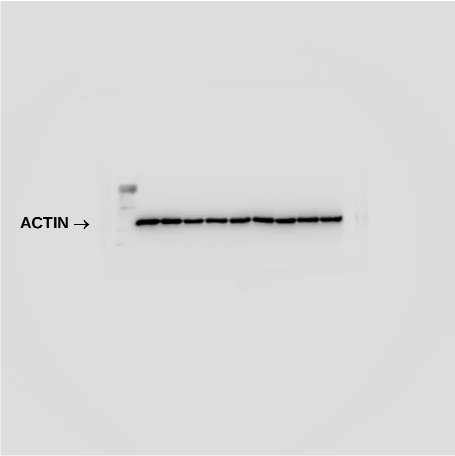


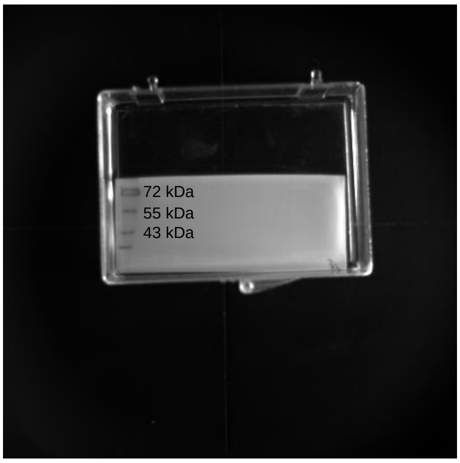


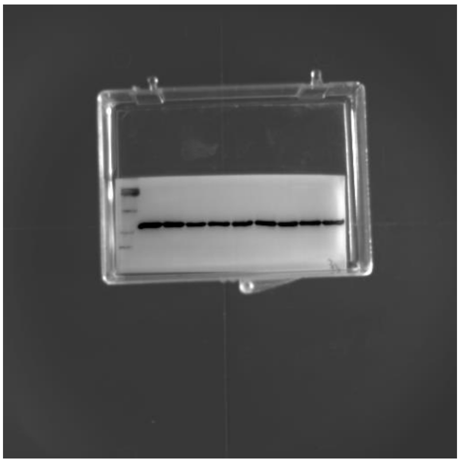

# original data – Western Blots N-CADHERIN

**chemiluminescence** | **blot photo** | **overlay**

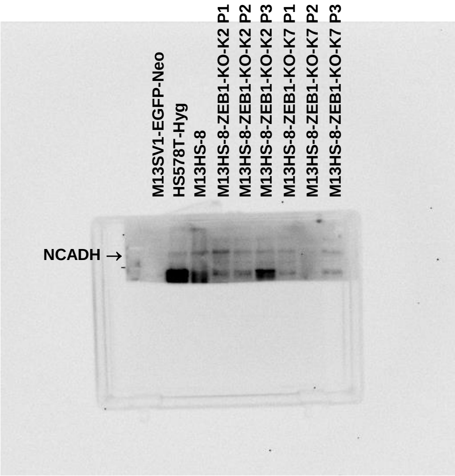


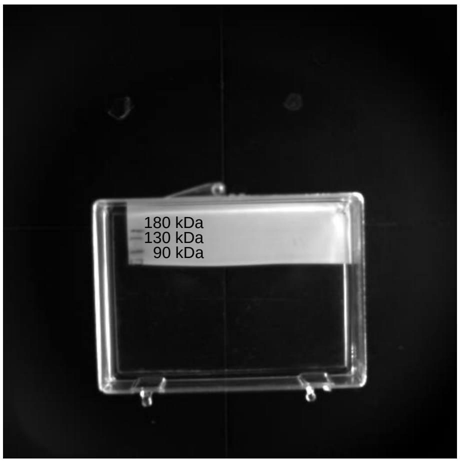


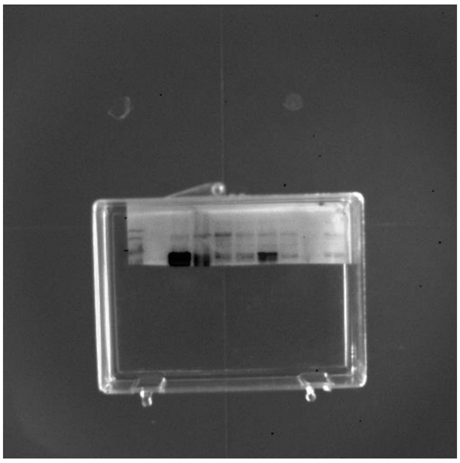

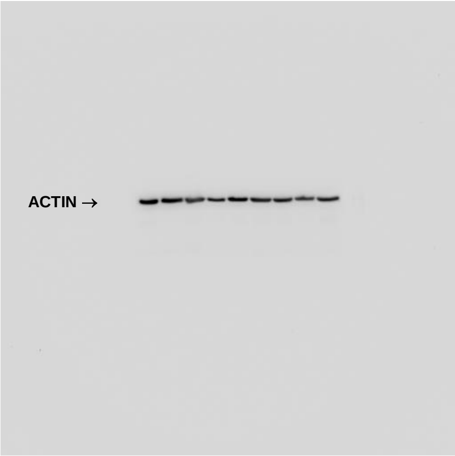


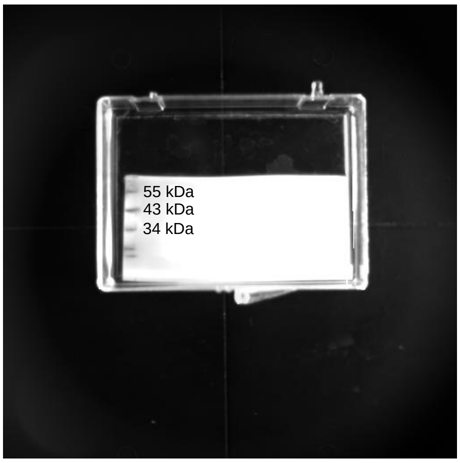


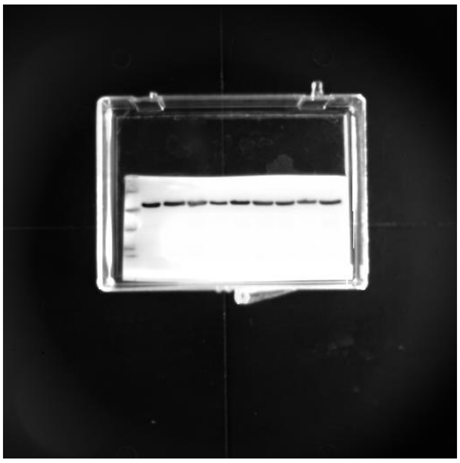

# original data – Western Blots VIMENTIN

| chemiluminescence | blot photo | overlay |
| --- | --- | --- |

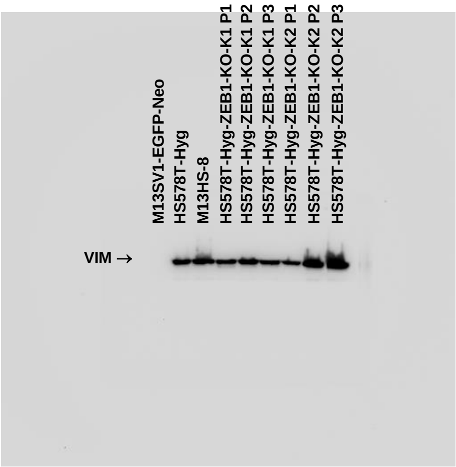


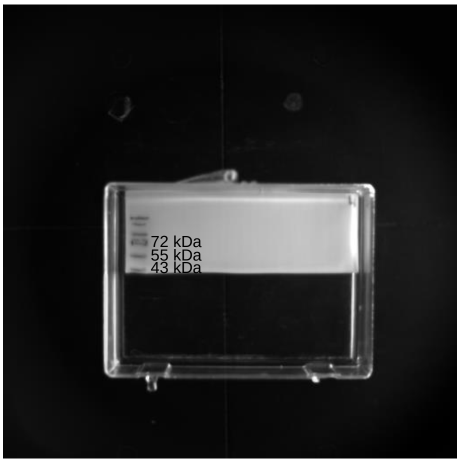


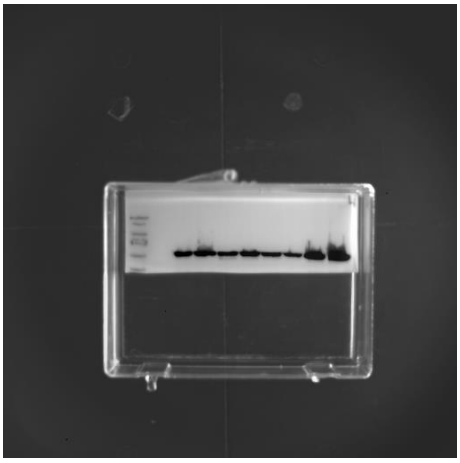

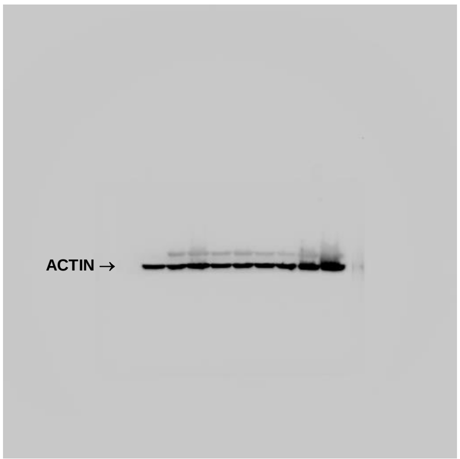


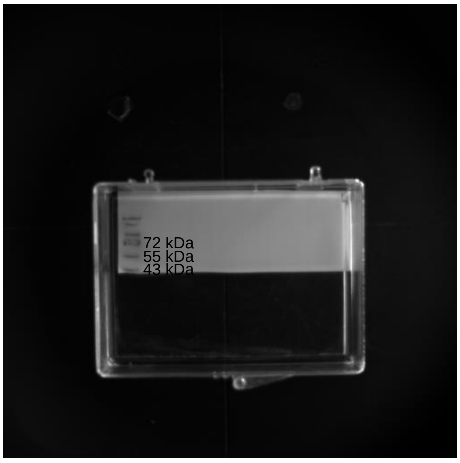


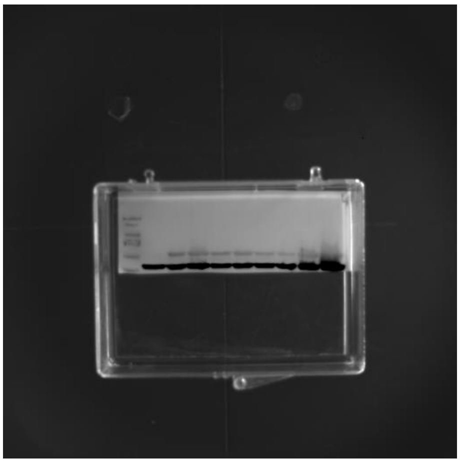

# original data – Western Blots VIMENTIN

## chemiluminescence | blot photo | overlay

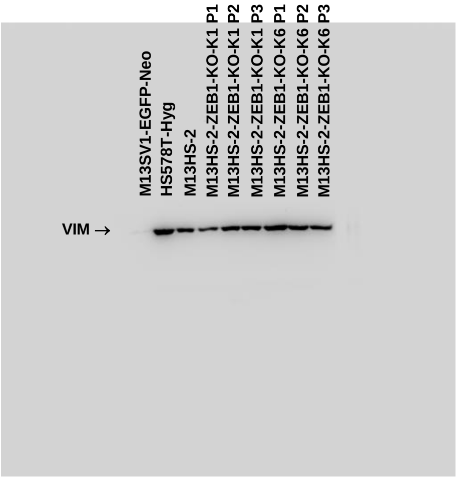


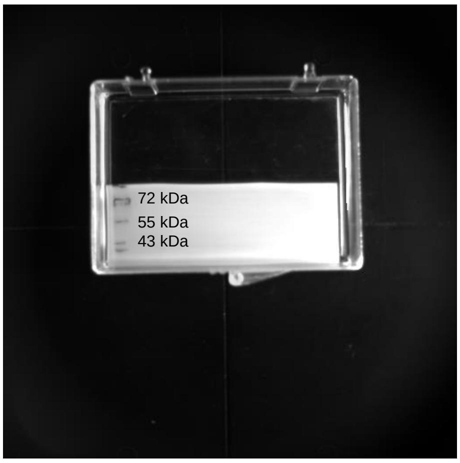


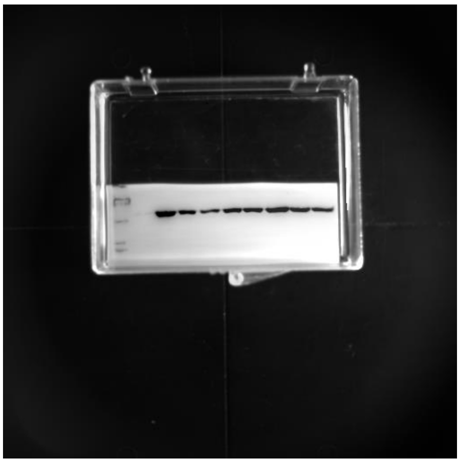

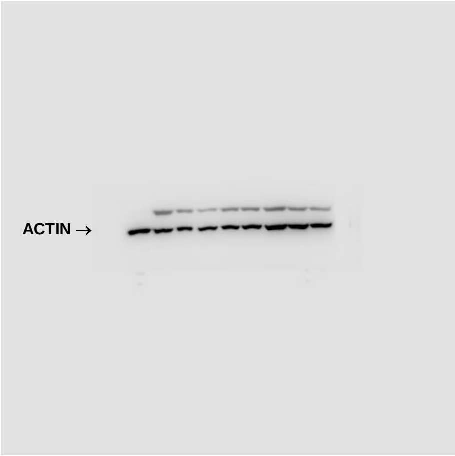


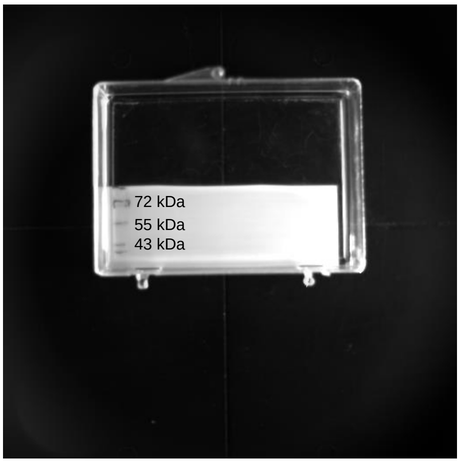


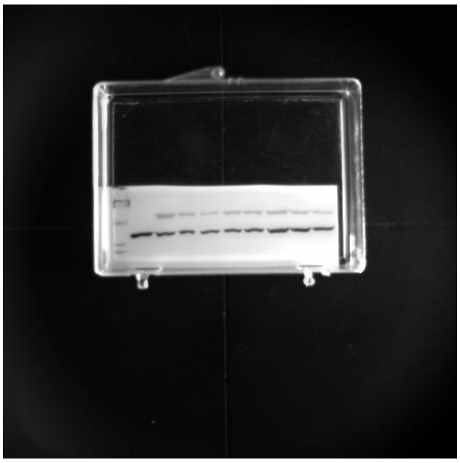

# original data – Western Blots VIMENTIN

**chemiluminescence** | **blot photo** | **overlay**

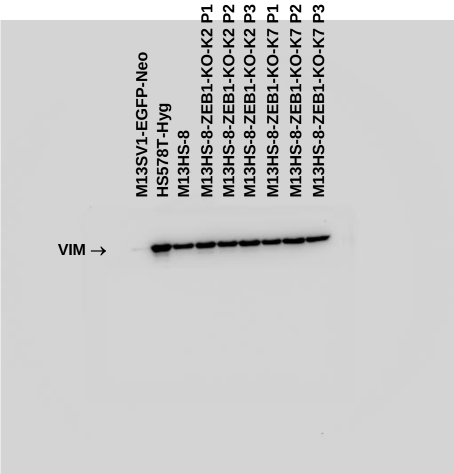


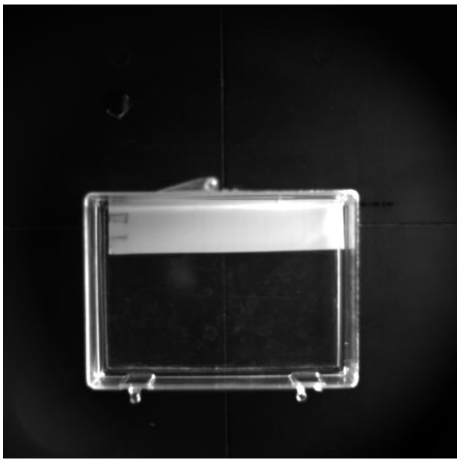

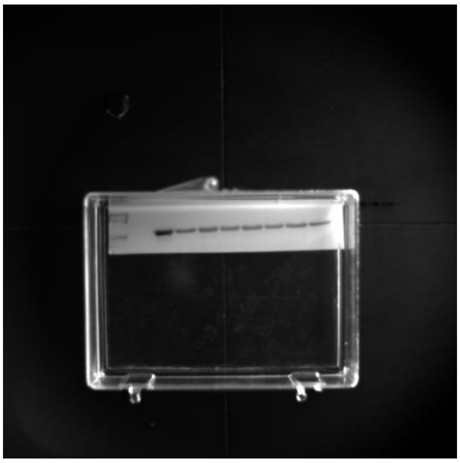

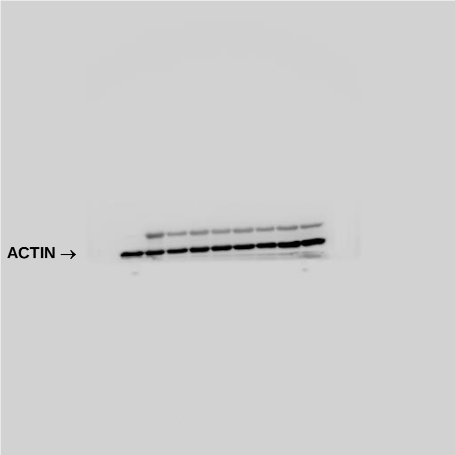


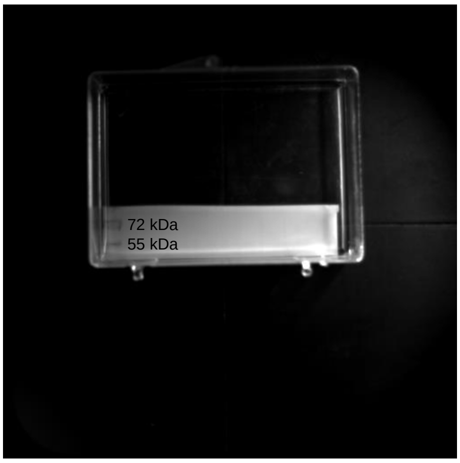


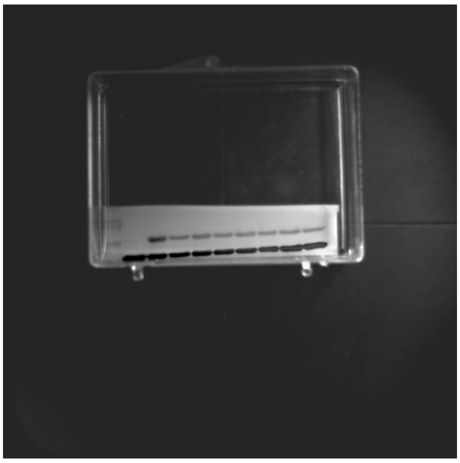

# original data – Western Blots WNT5A

**chemiluminescence** | **blot photo** | **overlay**

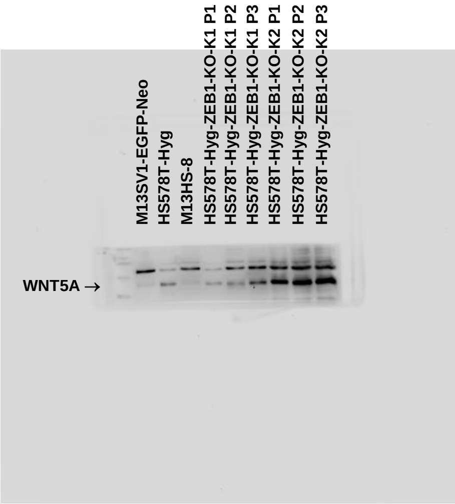


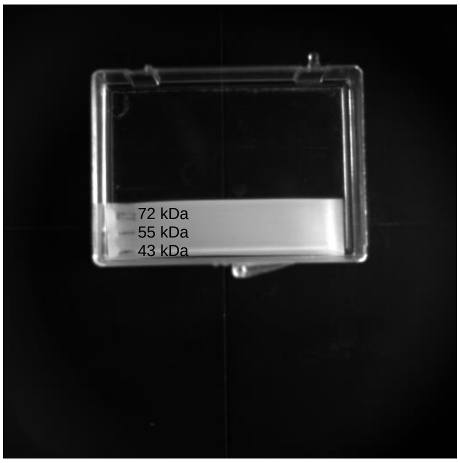


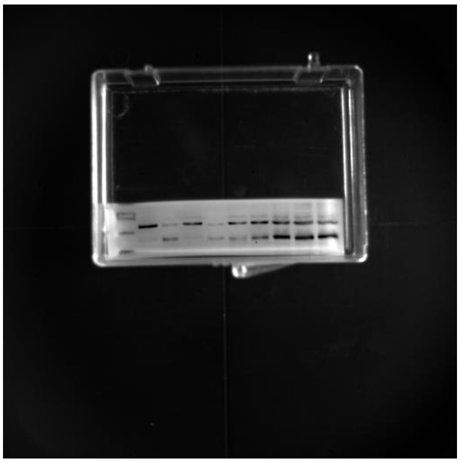

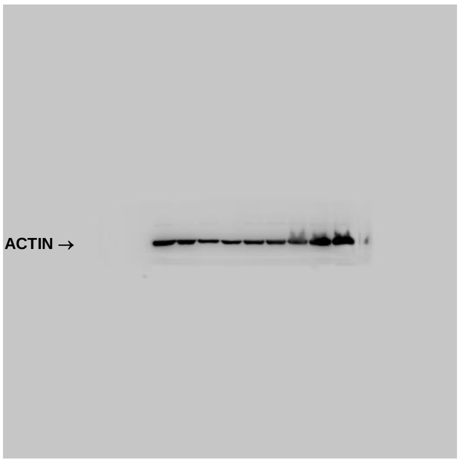


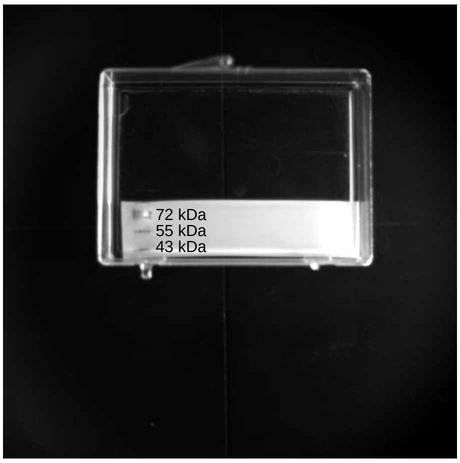


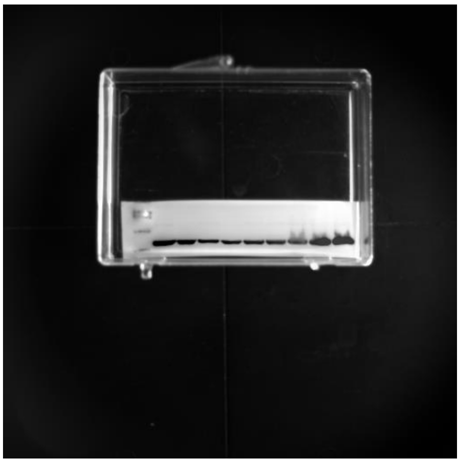

# original data – Western Blots WNT5A

**chemiluminescence** | **blot photo** | **overlay**

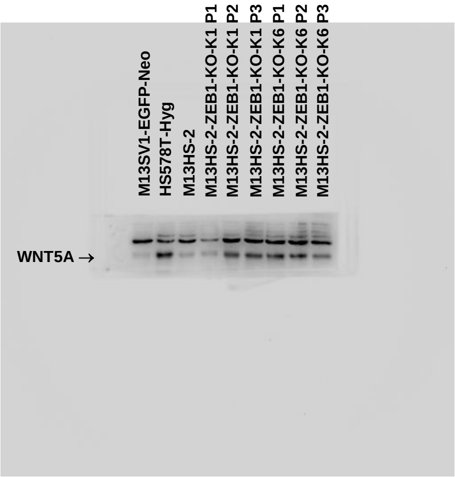


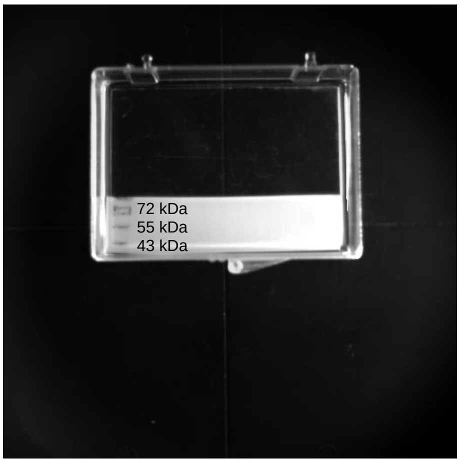


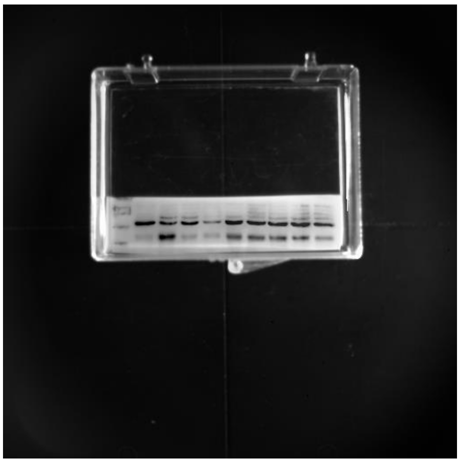

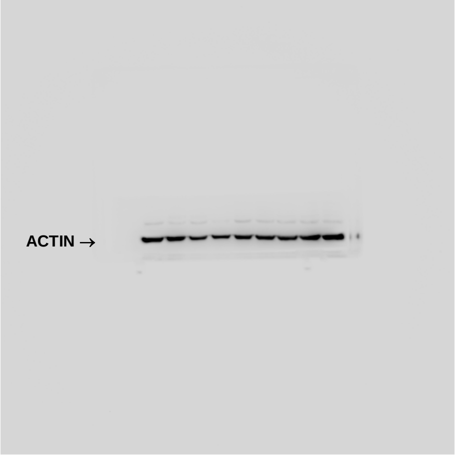


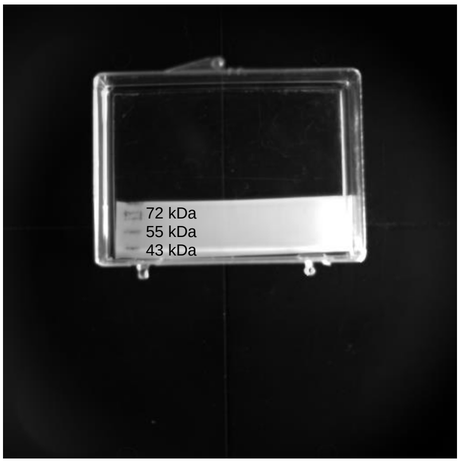


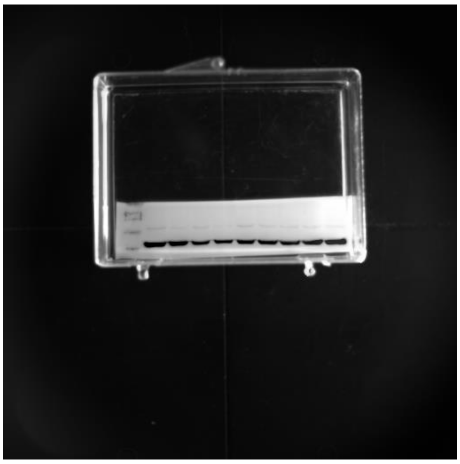

# original data – Western Blots WNT5A

**chemiluminescence** | **blot photo** | **overlay**

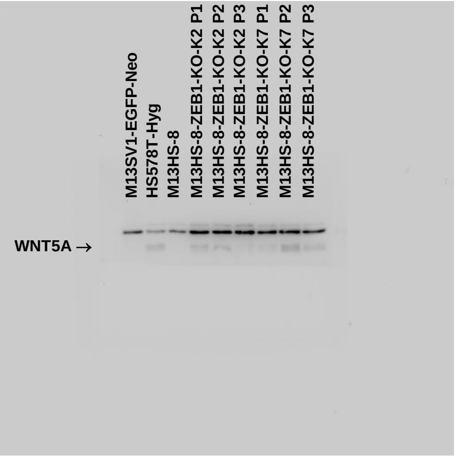


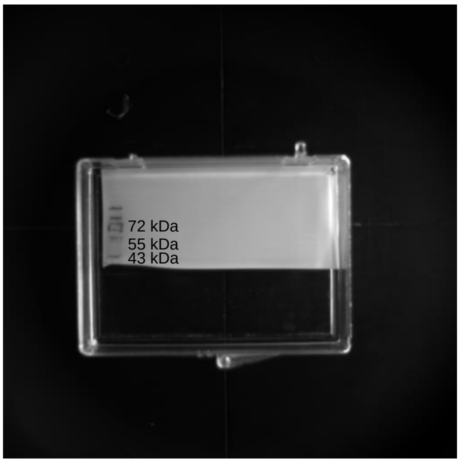


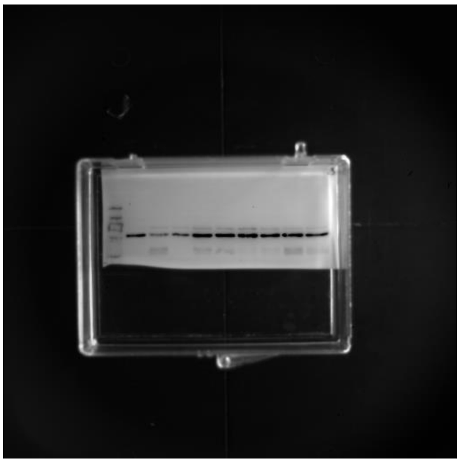

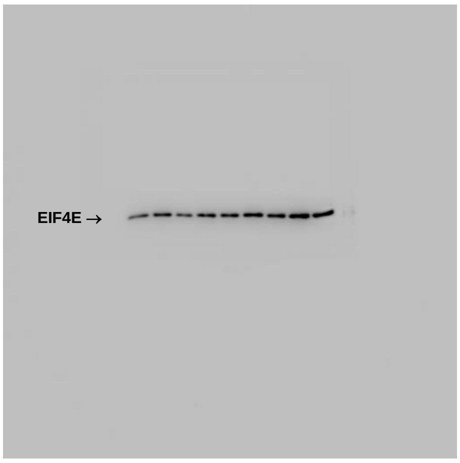


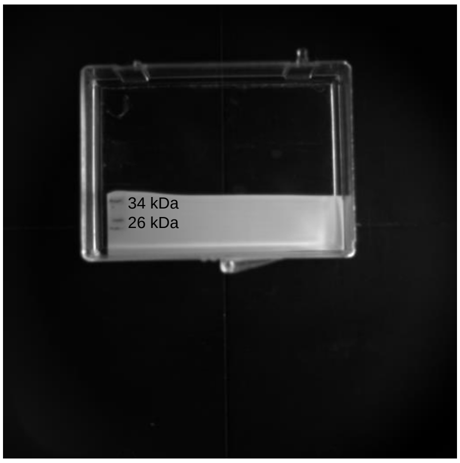


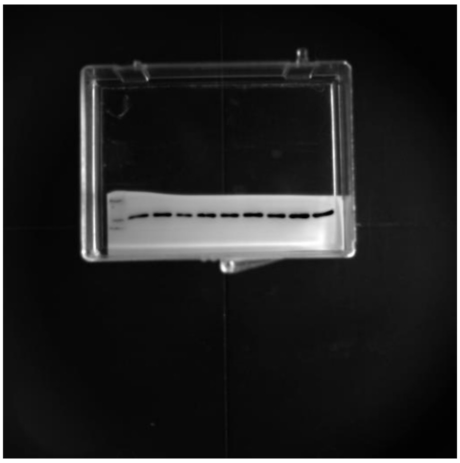

# original data – Western Blots ZEB2

**chemiluminescence** | **blot photo** | **overlay**

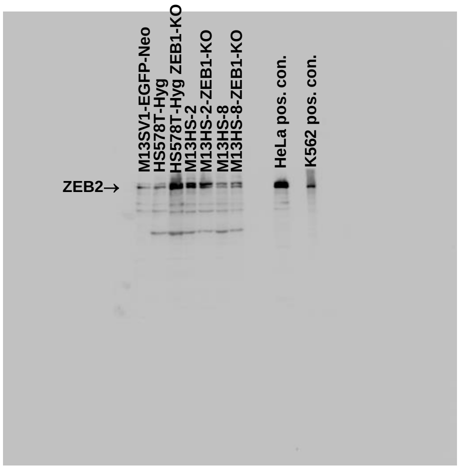


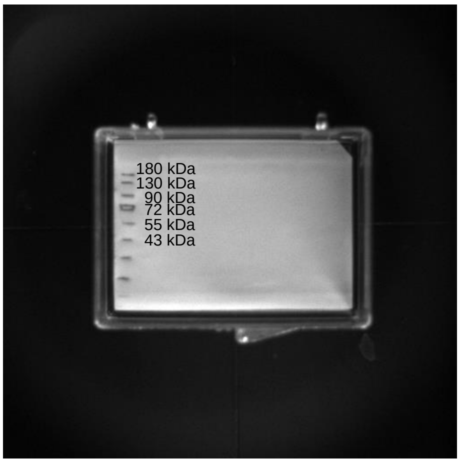


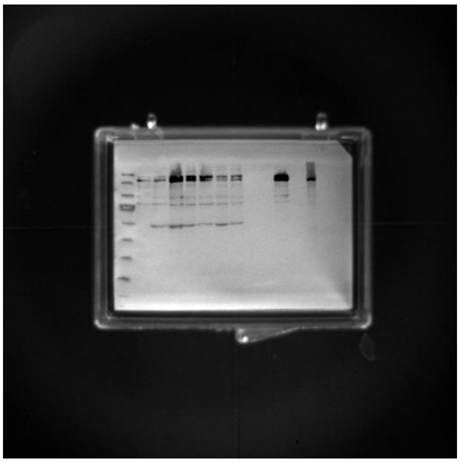

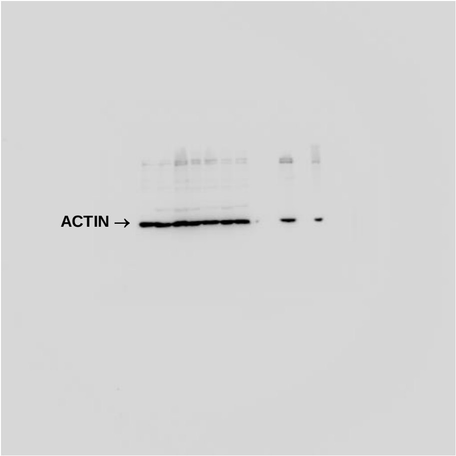


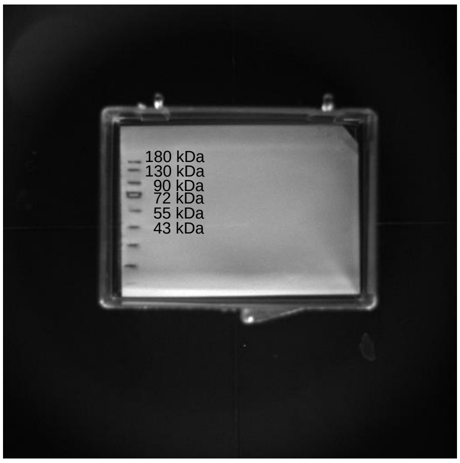


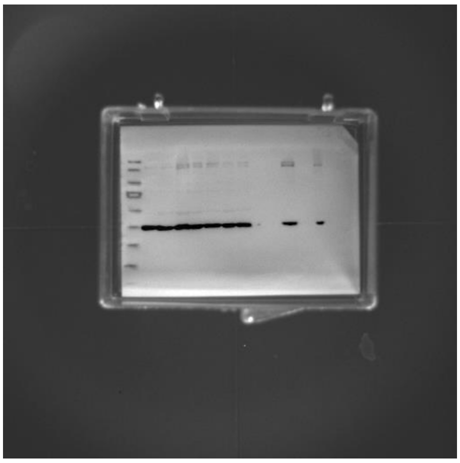

Supplement: Supplementary file 1 [file ijms-24-17310-s001.zip › ijms-2718011-supplementary.pdf]
